# The German Chemical Society's perspective on international collaboration with China

**DOI:** 10.1093/nsr/nwab017

**Published:** 2021-02-01

**Authors:** Peter R Schreiner

**Affiliations:** Professor of Organic Chemistry, Justus Liebig University Giessen, Germany; President 2020 & 2021 of the German Chemical Society

International collaboration has a long tradition amongst chemists worldwide, and I will take the liberty of starting with a personal note. My scientific forefather of the early nineteenth century, Justus von Liebig, went for a research stay in Paris/France (with J. L. Gay-Lussac) and later hosted at the University of Giessen more than 100 visiting scientists from many countries. It seems a trademark of excellent scientists that they recognize that science has no borders, and that the challenges we face can best be met when working together. This also applies to modern societies and exerting control over the global pandemic provides ample proof for this statement.

The German Chemical Society (GDCh), with its more than 30 000 members and 27 divisions, has been supporting international collaboration from the very beginning in all areas of chemistry. Scientific conferences, publications and the exchange of young as well as senior scientists play major roles. GDCh is a key partner within the European Chemical Society (EuChemS), promotes multi- and bilateral international scientific exchange and maintains cooperation agreements with 11 leading chemical societies worldwide, among them the American Chemical Society (ACS) and the Chinese Chemical Society (CCS). Even though the pandemic has led to cancellation of many events and exchange programs, GDCh members are working together with their colleagues worldwide using all available online tools. GDCh's long-lasting collaboration with many sister societies around the globe paved the way for profound scientific collaborations even under the currently difficult circumstances. Today, it pays off that GDCh and CCS had already established a close cooperation in 2004 when a GDCh delegation visited Beijing for the first time. Since then, GDCh and CCS have been organizing the ‘Sino-German Frontiers of Chemistry Symposium’ for young group leaders every other year either in China or in Germany. With financial support from the Sino-German Center for Research Promotion, seven meetings have been held to date in Beijing, Shanghai, Berlin, Munich, and Kloster Seeon, offering rich interdisciplinary scientific programs as well as cultural and social activities. Many of the more than 300 former participants have reached high scientific positions over time and still work together with international colleagues and friends from the former exchange. The last meeting planned for September 2020 in Shenzhen had to be postponed because of the pandemic, but will hopefully take place in 2022.

In 2008, together with the chemical societies from Great Britain, Japan, and the United States, GDCh and CCS established the ‘Chemical Sciences and Society Symposia (CS3)’. Supported by the chemical societies and the research funding agencies from all participating countries, the CS3 brings together leading researchers to discuss how the chemical sciences can help tackle some of the most daunting challenges that our world faces. Eight CS3 symposia have been held since 2009, rotating in the participating countries and always tackling topics around sustainability such as water resources, human health, rare elements, solar energy and sustainable plastics. Two meetings took place in China, in 2011 in Beijing and in 2017 in Dalian. The corresponding white papers from each symposium helped scientists, research funders and policy makers to identify and shape future research areas. CS3 is another example of how international collaboration in chemistry contributes to reaching many of the United Nations’ Sustainable Development Goals (UN SDGs).

International and trans-disciplinary collaboration is essential for the development of chemistry and all scientific areas. I am happy that the bilateral cooperation agreement between GDCh and CCS was renewed on the occasion of GDCh's 150-year anniversary celebrations in 2017 in Berlin. I am convinced as well as optimistic that after the pandemic we will see a new bloom of collaborations and personal friendships between researchers in our two countries and worldwide. The chemical societies are here to help in these endeavors.

